# Low Reactive Level Laser Therapy for Mesenchymal Stromal Cells Therapies

**DOI:** 10.1155/2015/974864

**Published:** 2015-07-26

**Authors:** Toshihiro Kushibiki, Takeshi Hirasawa, Shinpei Okawa, Miya Ishihara

**Affiliations:** Department of Medical Engineering, National Defense Medical College, 3-2 Namiki, Tokorozawa, Saitama 359-8513, Japan

## Abstract

Low reactive level laser therapy (LLLT) is mainly focused on the activation of intracellular or extracellular chromophore and the initiation of cellular signaling by using low power lasers. Over the past forty years, it was realized that the laser therapy had the potential to improve wound healing and reduce pain and inflammation. In recent years, the term LLLT has become widely recognized in the field of regenerative medicine. In this review, we will describe the mechanisms of action of LLLT at a cellular level and introduce the application to mesenchymal stem cells and mesenchymal stromal cells (MSCs) therapies. Finally, our recent research results that LLLT enhanced the MSCs differentiation to osteoblast will also be described.

## 1. Introduction

Mesenchymal stromal cells (MSCs) are the promising source for the regenerative medicine and repair of various tissues in the treatment of a range of diseases. The differentiation of these cells to different lineages is dictated by the local extracellular matrix (ECM) as well as spatial and temporal cues, including growth factors and cell-cell interactions. In bone formation, mechanotransduction and physical cues, such as shear stress and fluid flow [1], also influence the differentiation of MSCs. However, the fundamental questions that how to control the differentiation of MSCs to matured cells must be answered. In particular, a better understanding of how specific factor may alter the fate of differentiation of MSCs is needed. Their rapid and selective differentiation should provide the potential of new therapeutic approaches for the restoration of damaged or diseased tissue. We have reported that the laser irradiation to MSCs influences cell differentiation and possible mechanisms of cells differentiation by laser were proposed [2–4]. In this review, we will describe the mechanisms of action of laser irradiation and introduce the application to MSCs therapies including our research results.

A laser (light amplification by stimulated emission of radiation) is a device that generates electromagnetic radiation that is relatively uniform in wavelength, phase, and polarization. This technology was originally described by Maiman in 1960 in the form of a ruby laser [5]. The properties of lasers have allowed for numerous medical applications, including their use in surgery, activation of photodynamic agents, and various ablative therapies in cosmetics, all of which are based on heat generated by the laser beam, in some cases leading to tissue destruction [6–13]. Low reactive level laser therapy (LLLT) is a form of medical treatment in which human tissue is irradiated with a low-powered laser (on the order of several hundred milliwatts) to induce therapeutic changes. In an attempt to explore the carcinogenic potential of laser light, Mester et al. in 1967 applied a low-powered ruby laser with a 694 nm wavelength to the shaved dorsal skin of mice [14]. Contrary to their expectations, the laser irradiation did not cause cancer but instead improved hair growth. As the first study to document the biological effect of lasers, their findings became a springboard for subsequent LLLT research. Although light-based therapies had been used for a long time and ultraviolet therapy has a history longer than a century [15], the work of Mester et al. was significant in demonstrating the effects of laser light, which has the unique characteristics of monochromaticity and coherence. Following subsequent experiments, Mester and colleagues reported in 1971 that low power laser rays accelerated wound healing [16]. Since those early days, numerous* in vitro* and* in vivo* studies of LLLT in the context of regenerative medicine have demonstrated a wide variety of therapeutic effects including improvements in wound healing, collagen synthesis, cell proliferation, fracture repair, and local blood circulation, as well as suppression of inflammation and pain. According to da Silva et al. [17], the types of laser most frequently used for wound healing and tissue repair are helium-neon (He-Ne) lasers and diode lasers, including gallium-aluminum-arsenic (Ga-Al-As), arsenic-gallium (As-Ga), and indium-gallium-aluminum-phosphide (In-Ga-Al-P) lasers.

A large number of literatures and review articles [18–20] have shown that LLLT accelerates wound healing, and we present some typical results here. Irradiation of cultured human keratinocytes with a 632 nm helium-neon laser elevated the interleukin-1*α* and interleukin-8 mRNA levels, promoted keratinocyte migration and proliferation, and accelerated wound repair [21]. In addition,* in vitro* studies of laser-irradiated cells revealed elevated levels of vascular endothelial growth factor (VEGF) [22] and transforming growth factor *β* (TGF *β*) expression [23]. These findings illustrate the laser-enhanced expression of many cytokines and growth factors in keratinocytes and fibroblasts, the key cellular mediators of the wound-healing process. In addition, after mice with lipopolysaccharide-induced peritonitis were irradiated with a 904 nm gallium arsenide (Ga-As) laser, inflammatory cell migration was inhibited [24]. In a rat model of carrageenan-induced pleuritis, a 660 nm In-Ga-Al-P laser suppressed the production of inflammatory cytokines and the migration of inflammatory cells [25]. A group of researchers led by Albertini are actively pursuing research on LLLT's anti-inflammatory effects [26–46]. In the field of regenerative medicine, LLLT accelerates osteoblast proliferation, bone formation [47], and bone repair [48]. Various groups have suggested the involvement of insulin-like growth factor-1 (IGF-1) [49], mitogen-activated protein kinase (MAPK)/extracellular signal-regulated kinase (ERK) [50], and bone morphogenetic protein (BMP)/Smad signaling cascades [51]. In addition, LLLT confers physiological effects to regeneration of damaged neurons [52–55], articular cartilage [56], and muscle tissue [57–59]. To date, several mechanisms of biological action have been proposed, although none have been clearly established. These include augmentation of cellular ATP levels [60–62]; manipulation of inducible nitric oxide synthase (iNOS) activity [63–67]; suppression of inflammatory cytokines such as tumor necrosis factor- (TNF-) *α* [61, 68–70], interleukin- (IL-) 1*β* [27, 70, 71], IL-6 [25, 70, 72–74], and IL-8 [25, 70, 72, 75]; upregulation of growth factors such as PDGF, IGF-1, NGF, and FGF-2 [71, 76–78]; alteration of mitochondrial membrane potential [79–82] due to chromophores found in the mitochondrial respiratory chain [83–85]; stimulation of protein kinase C (PKC) activation [86]; manipulation of nuclear factor- (NF-) *κ*B activation [28]; induction of reactive oxygen species (ROS) [87, 88]; modification of extracellular matrix components [89]; inhibition of apoptosis [79]; stimulation of mast cell degranulation [90]; and upregulation of heat shock proteins [91].

In the following paragraphs, we will discuss the cellular effects of LLLT that underlie its biological actions. Through our research, we have discovered (i) the presence of intracellular photoreceptors and physiological changes resulting from photoreception, (ii) postirradiation modifications in cellular signal transduction cascades, and (iii) postirradiation alterations in gene expression. These various effects do not occur in an isolated manner. Here, we will focus on how these effects interact with each other to induce modifications in cellular functions. We will also summarize typical results of the LLLT application to MSCs therapies.

## 2. Laser-Induced Cellular Responses

In order to elucidate the biological mechanisms underlying effects of low power lasers documented in experimental and clinical studies, one must consider the cellular responses to laser irradiation. The photons must be absorbed by electronic absorption bands belonging to some molecular chromophores or photoreceptors [92]. A chromophore or photoreceptor is a molecule (or part of a molecule) where the energy difference between electrons in two different molecular orbitals falls within the energy possessed by photons in the visible spectrum. In this section, we describe the intracellular photoreceptors and the cellular responses to laser light. One of the most distinctive features of LLLT relative to other modalities is that the effects are mediated not through induction of thermal effects but rather through a process called “photobiostimulation.”

### 2.1. Intracellular Photoreceptor

In photobiology, photoreception refers to the intracellular process whereby wavelength-specific photoreceptors absorb photon energy [92]. Photoreceptors are biomolecules that are capable of absorbing photoenergy, either intrinsically or via a molecular component. The mitochondrial respiratory chain includes multiple photoreceptors, as described below.

#### 2.1.1. Cytochrome *c* Oxidase

The enzyme cytochrome *c* oxidase receives electrons from respiratory-chain substrates via the cytochrome pathway and transfers them to oxygen molecules. Cytochrome *c* oxidase has been proposed as the endogenous photoreceptor in the visible to near-infrared region (above 600 nm) [93]. Scientists have conducted extensive research on the photobiomodulation by cytochrome *c*oxidase, particularly in neuronal cells. In a study of neurons functionally inactivated by tetrodotoxin, a voltage-dependent sodium channel blocker [94], near-infrared irradiation restored the activity of intoxicated cytochrome *c* oxidase by altering its redox state. In another study, laser irradiation of mitochondria increased cytochrome *c* oxidase activity, polarographically measured levels of oxygen uptake, and subsequent ATP production [95]. Many other* in vitro* and* in vivo* studies of laser-induced cell growth have reported changes in cytochrome *c* oxidase activity and ATP production following irradiation [81, 96–103].

#### 2.1.2. Porphyrin

Porphyrins are a group of macrocyclic organic compounds that contain four pyrrole subunits joined by methine bridges. These mostly green- or red-colored compounds have specific absorption spectra and emit red fluorescence. Naturally occurring porphyrins, including those found in the human body, often form complexes with an iron or magnesium ion coordinated to the four pyrrole nitrogen atoms. For example, iron protoporphyrin IX (PPIX) complexes (i.e., heme* b*) form the prosthetic groups of hemoglobin, catalase, and peroxidase. Mitochondrial cytochromes also contain iron-porphyrin groups (nonheme* b*). The PPIX absorption spectrum has five major peaks in the range of 400 to 650 nm, with peak height decreasing as the absorption wavelength increases. The excited triplet state of PPIX, formed by absorption of laser photons, generates ROS by transferring energy to ground-state oxygen atoms. A mode of photodynamic therapy (PDT) that exploits this feature has been developed for anticancer treatment. In this technique, patients are administered PPIX or its precursor, 5-aminolevulinic acid (ALA), and ROS are generated with local laser irradiation to kill malignant cells or epithelial cells of vascular neoplasms [104].

#### 2.1.3. Flavoproteins (Flavin Proteins)

Flavoproteins are a group of protein complexes containing a riboflavin prosthetic group (e.g., flavin adenine dinucleotide [FAD] or flavin mononucleotide [FMN]). Most flavoproteins function as flavin enzymes, which use iron, molybdenum, copper, manganese, and other heavy metal ions as cofactors. These proteins have major absorption peaks in the range of 350 to 500 nm. Flavoproteins mediate a wide array of biological processes, such as bioluminescence, quenching of oxidative stress-induced radicals, DNA repair, and apoptosis [105]. Some researchers, including the present author, have reported the roles of flavoproteins as intracellular photoacceptors [2, 3, 106].

#### 2.1.4. Other Groups of Photoreceptors

In addition to the three major groups of photoreceptors explained above, there are other types of photoreceptors, including rhodopsin, bilirubin, melanin, pterin, vitamin B6, vitamin K, nicotinamide adenine dinucleotide (phosphate) hydrogen [NAD(P)H], urocanic acid, and tryptophan.

### 2.2. Laser-Induced Changes in Signaling Cascades

It is clear that signal transduction pathways regulate cells in order to transduce the signal from the cellular photoreceptors that absorb photon energy to the biochemical machinery that controls gene transcription. Many researchers believe that the photon energy captured by intracellular receptors leads to alterations in gene and protein expression via a series of processes that modify signaling cascades. However, little is known regarding how light-stimulated receptors transduce their signals to the nucleus, or how these signals mediate the expression of particular genes. We have studied the mechanisms underlying the promotion and suppression of stem cell differentiation, with a focus on FAD-containing cryptochromes as cellular photoreceptors [2, 3]. Our research suggested that light-activated cryptochromes migrate into the nucleus, where they regulate the expression of proteins located downstream of the E-boxsequence. As a matter of course, cell functions are regulated by an array of other factors, including ROS. Therefore, we will now describe the biochemical changes LLLT induces beyond the photoreceptor absorption of light energy, as reported in the literature.

#### 2.2.1. Redox Pathways

Several oxygen and nitrogen radicals have been proposed to transduce mitochondrial signals to the nucleus. Those species react with NAD, NADH, NADP, NADPH, glutathione, glutathione sulfide, thioredoxin, and thioredoxin sulfide [107]. The cell contains several endogenous sensors for these species (typically, superoxide dismutase [SOD]) [108]. Upon detection of ROS, the cell activates self-defense pathways by altering its gene expression patterns [109]. If these self-defense mechanisms fail, the cell will undergo apoptosis. The levels of ROS strictly determine the expression of proteins regulating cell proliferation, suggesting that oxygen radicals act as second messengers [110, 111]. ROS are considered to play key roles in the control of cellular functions [112]. Low power laser beams with wavelengths around 630 nm generate oxygen radicals in exposed cells [113, 114]. We have also discovered significant increases in the levels of oxygen radicals in cells exposed to laser light (wavelength: 405 nm) [87]. Although the specific mechanism remains unknown, laser-induced intracellular generation of ROS probably involves energy transfer from PPIX and other photoreceptors present in the cell. In addition, several groups have described cellular functions mediated by nitric oxide (NO), which is upregulated by laser irradiation, as well as by inducible nitric oxide synthase (iNOS) [65, 67, 114–116]. The mechanism of laser-induced control of cellular functions is believed to hinge on the regulation of photoreceptor activity and the intracellular levels of ROS.

#### 2.2.2. Transcription Factors

Several researchers have reported that the aforementioned redox pathways trigger changes in the expression of many transcription factors. Here, we briefly describe one of the best-characterized transcription factors in the LLLT field, NF-*κ*B [117, 118]. Published articles on other transcription factors mediating a multitude of cell functions have made it clear that their expression levels are also modified upon exposure to laser irradiation. As a transcription factor, NF-*κ*B can simultaneously induce the expression of IL-1, IL-2, IL-6, IL-8, IL-12, TNF-*α*, and other proinflammatory cytokines. It also controls the expression of apoptosis-related proteins, which play a critical role in tumor cell growth and immortalization. Several studies have shown that the aforementioned redox pathways trigger increases in NF-*κ*B levels [117, 118]. This mechanism is considered to account, at least in part, for the observation that low power laser irradiation induces the expression of various cytokines. Rizzi et al. have showed that histological abnormalities with increase in collagen concentration and oxidative stress were observed after trauma. The associated reduction of inducible nitric oxide synthase overexpression and collagen production suggest that the NF-*κ*B pathway is a signaling route involved in the pathogenesis of muscle trauma [118]. The hypoxia-inducible factor (HIF-1) is also a ubiquitous transcription factor involved in the control of cell and tissue responses to hypoxia, specifically in angiogenesis, hematopoiesis, and anaerobic energy metabolism. There are over 70 genes which have been established as direct targets by identification of critical HIF-1 binding sites [119]. In addition, the activator protein- (AP-) 1 is involved in cellular proliferation, transformation, and death [120]. AP-1 is not a single protein but a complex array of heterodimers composed of proteins that belong to the Jun, Fos, and ATF subfamilies, which recognize specific nuclear target sequences. Different dimeric combinations can stimulate a variety of gene expression patterns. AP-1 can be activated by growth factors, cytokines, hypoxia, ionizing, and UV radiation [121, 122].

#### 2.2.3. Circadian Rhythm

The circadian rhythm, a roughly 24-hour cycle of cellular events, was acquired during the early stages of evolution and is ubiquitous from unicellular organisms to mammals. Several mammalian clock genes work together to establish a stable oscillation of approximately 24 hours. Circadian clock proteins, such as brain-muscle Arnt-like protein 2 (BMAL2), clock, cryptochrome (CRY), and period (PER), set the pace of the clock in almost all cell types (e.g., the timing of cell division and other cellular activities). CRY, a blue-light receptor in higher plants and Drosophilidae [123], utilizes as its chromophore the FAD coenzyme, which undergoes blue-light excitation. This observation led to the idea that light-excited FAD transfers electrons to a certain substrate. However, the validity of this theory has not been tested. Bone metabolism (remodeling) is a continuous homeostatic process involving resorption of existing bone by osteoclasts and formation of new bone by osteoblasts. Fu et al. showed that circadian rhythms mediate bone formation [124], and Kawasaki et al. reported that the E-box motif, a circadian regulatory sequence, is involved in the osteoblast expression of bone morphogenetic protein- (BMP-) 4 [125]; these findings indicate that CRY proteins regulate various homeostatic and physiological events via E-box elements. We conducted research on the effects of lasers on endocellular distribution and expression of CRY using laser beams (wavelength: 405 nm), which correspond to the absorption band of the CRY coenzyme FAD [2]. We will describe the results below.

## 3. LLLT for MSCs Therapies

**Figure 1:**
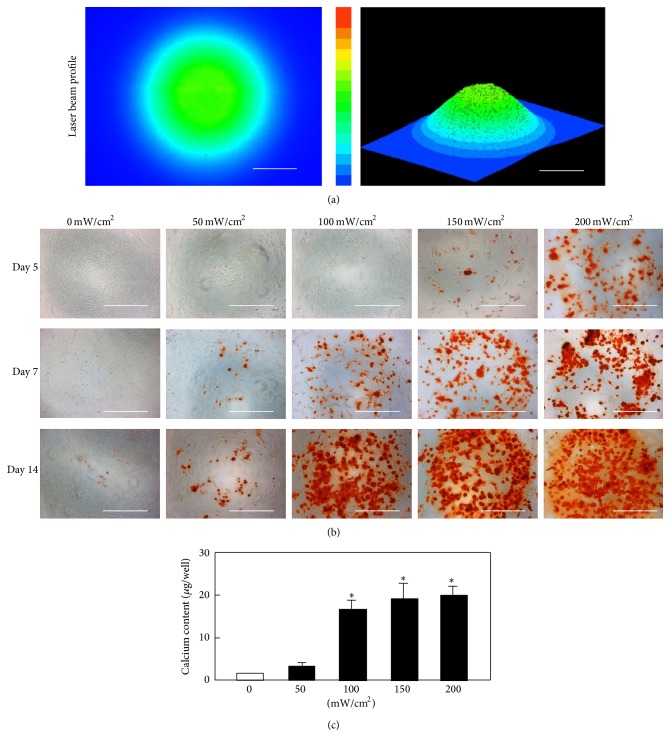
(a) The beam profile of the blue laser (wavelength: 405 nm) used in this study (scale bars: 200 *µ*m). MSCs were irradiated for 180 sec at various laser power levels. (b) Alizarin red S staining of irradiated MSCs (magnification 50x; scale bars: 400 *µ*m). After laser irradiation, calcium deposition had increased around the cells in a dose-dependent manner. (c) The quantitative calcium content increased after blue laser irradiation (day 14) relative to nonirradiated cells. Calcium content increases varied with laser energy level (^∗^ *P* < 0.01, indicating significant difference between the calcium content of laser-irradiated MSCs and controls) [3].

**Figure 2:**
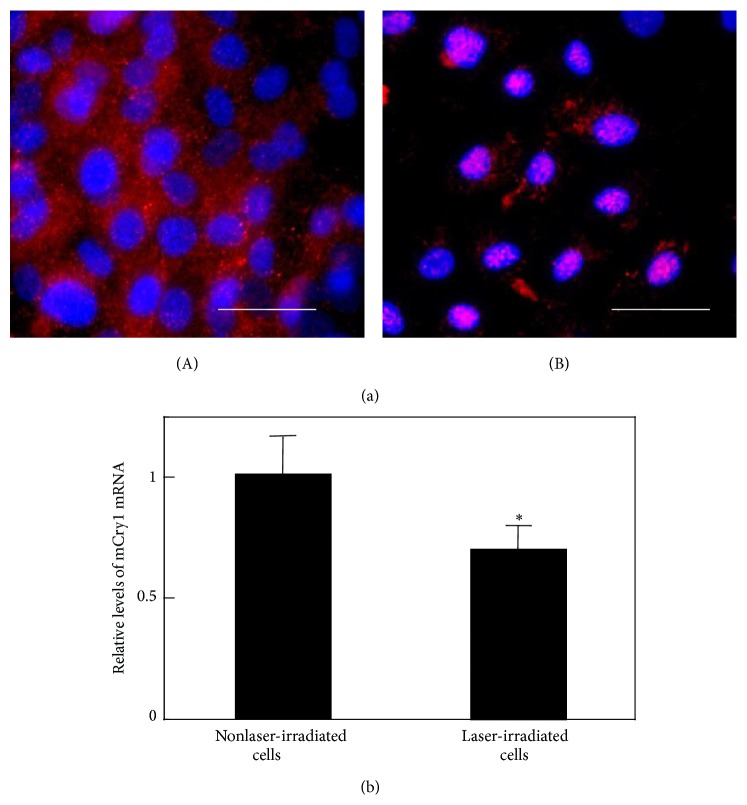
(a) Subcellular location of CRY1 proteins in MSCs after laser irradiation (200 mW/cm^2^). Cells were double-labeled with DAPI (blue) and CRY1 (red). CRY1 localized to the cytoplasm prior to laser irradiation (A). However, after laser irradiation, CRY1 translocated to the nucleus (B) (scale bars: 50 *µ*m). (b) mRNA levels of Cry1 in MSCs 24 h after laser irradiation (200 mW/cm^2^) and in nonirradiated cells. Samples were normalized to mRsp18. The mRNA levels of Cry1 decreased after blue laser irradiation relative to nonirradiated cells (^∗^ *P* < 0.01, indicating significant difference between the relative mRNA levels of laser-irradiated MSCs and controls) [3].

Since LLLT has been scientifically proven as a beneficial therapeutic modality for numerous diseases and diseased conditions, it was applied to enhance MSCs proliferation and differentiation. The recent 3-year reports regarding LLLT application to increase MSCs proliferative and differentiation potential were summarized in Table 1 [126–135, 138–143]. Abrahamse's group published some literatures for LLLT application to stem cells. It is the cellular effect of increasing proliferation and viability that may significantly contribute to the addition of LLLT to the many biomedical disciplines that further augment the successes of regenerative medicine [144]. They reported that low power laser irradiation has been shown to induce adipose-derived stem cell activity by increasing migration, proliferation, and viability, activating protein expression and inducing differentiation in progenitor cells [145–147]. Wu et al. reported that LLLT suppresses inflammatory response of human adipose-derived stem cells by modulating intracellular cyclic AMP level and NF-*κ*B activity [129]. Lipopolysaccharide- (LPS-) induced proinflammatory cytokine expression was inhibited by LLLT and the intracellular cAMP level, which acts to downregulate NF-*κ*B transcriptional activity which was increased. Those results indicate that LLLT can potentially be applied in anti-inflammatory therapy followed by stem cell therapy. We reported that the laser irradiation can direct the extracellular calcification of primary MSCs by altering the intracellular localization of the circadian rhythm protein, CRY1 [2, 3].  Figure 1 presents the beam profile of the laser (wavelength: 405 nm) used in the study (Panel (a)) and the changes in mouse bone marrow mesenchymal stromal cells irradiated for 3 minutes and then cultured for 14 days in osteoblast differentiation medium (Panel (b)) [3]. Alizarin red staining revealed that the stained cells were distributed in a circular area with a diameter similar to that of the laser beam. In addition, the results of immunostaining for CRY1 protein are represented in Figure 2. Whereas CRY1 was distributed across the cytoplasm in control cells, it was localized to the nucleus in cells exposed to laser (wavelength: 405 nm) irradiation. The timing of nuclear accumulation of clock proteins constitutes an important step in the transcription-translation feedback loop driving the circadian core oscillator and is controlled by regulating protein localization and turnover. Our results show that these laser beams promote the nuclear localization of CRY1 and mediate the expression of CRY1 and other proteins downstream of the E-box, which played a critical role in deciding the expression of BMPs [3]. We also reported that laser irradiation suppressed the adipocyte differentiation of mesenchymal stromal cells [2] and accelerated their differentiation into chondrocytes [4]. Abramovitch-Gottlib et al. reported that the consequent phenotype modulation and development of MSCs towards ossified tissue were studied in the combined 3D biomatrix/LLLT system [148]. Their results obtained from the irradiated samples showed enhanced tissue formation, appearance of phosphorous peaks, and calcium and phosphate incorporation to newly formed tissue. Moreover, in irradiated samples ALP activity was significantly enhanced in early stages and notably reduced in late stages of culturing. Those findings of cell and tissue parameters up to 28 days of culture revealed higher ossification levels in irradiated samples compared with the control group. They suggested that both the surface properties of the 3D crystalline biomatrices and the LLLT have biostimulatory effect on the conversion of MSCs into bone-forming cells and on the induction of* ex vivo* ossification [148]. In addition, lasers in visible wavelength were used mostly for LLLT, but the novel laser sources, such as terahertz (THz) laser, were recently investigated for MSCs therapy [135–137]. Alexandrov et al. reported that extended exposure to broad-spectrum THz radiation results in specific changes in the functionality of cellular DNA. Certain genes in irradiated MSCs cultures are activated, while other genes are repressed. Many of the MSCs genes do not respond to the selected radiation conditions at all, showing that the effect is specific. Additionally, 9 hours of exposure causes significant changes in the MSCs gene expression, while the response to shorter duration (2 and 4 hours) is appreciably less pronounced. Hence, they discussed that the effect of THz radiation was gene and exposure specific and most likely is at the level of DNA transcription [137]. Although each researcher used a different type of laser (i.e., wavelength, power, and pulse-width), MSCs proliferative and differentiation potential can be increased. The mechanisms involved remain to be clarified, but LLLT is a valid approach for the preconditioning of MSCs* in vitro* prior cell transplantation.

## 4. Conclusion

Regenerative medicine and stem cell therapy have the potential to provide diseases-free, functional tissues and organs, improving the quality of life for patients. They have also the ability to transform the treatment of human disease by introducing combined innovative new therapies such as stem cell therapies and LLLT. Today, researchers are conducting intensive basic and clinical research in the area of laser medicine and photobiology, with the goal of developing new diagnostic and therapeutic modalities. Here, we described some of the latest advances in research on the cellular effects of irradiation with lasers to MSCs. The biological mechanisms underlying such responses significantly differ by the type of laser, target of cells, and other experimental conditions. With the appropriate use of LLLT, the proliferation rate of cultured cells, including MSCs, can be increased, which would be very useful in tissue engineering and regenerative medicine. We must accumulate a systematic knowledge base by carefully analyzing the experimental data currently available, as well as data collected in the future. We believe that light-based biomedical research will open new horizons for photodiagnosis, phototherapy, and MSCs therapies.

## Figures and Tables

**Table 1:** The effect of LLLT on the MSCs proliferation and differentiation (literatures published in recent 3 years).

Authors	Brief description	Reference
Park et al.	LLLT enhanced angiogenic effect of adipose-derived stromal cells (ASCs) spheroid in hind limb ischemia mice. LLLT is an effective biostimulator of spheroid ASCs in tissue regeneration that enhanced the survival of ASCs and stimulated the secretion of growth factors in the ischemic hind limb.	[126]

Farfara et al.	MSCs were stimulated by LLLT in order to affect neurological behavior and beta-amyloid burden in progressive stages of Alzheimer's disease mouse model.	[127]

Yang et al.	LLLT was applied as an adjunct therapy for MSCs transplantation on the functional recovery of crushed sciatic nerve in rats.	[128]

Wu et al.	LLLT increased the intracellular level of cAMP, which acts to downregulate NF-*κ*B transcriptional activity.	[129]

Nagata et al.	The combination of bone marrow aspirate/LLLT yielded significantly greater bone formation in surgically created critical-size defects in rat calvaria.	[130]

Manuguerra-Gagné et al.	A laser-induced model of open angle glaucoma (OAG) was used to evaluate the potential of bone marrow cell populations and the mechanisms involved in tissue repair. Laser-induced tissue remodeling as a method of targeting effector cells into damaged tissues was also evaluated.	[131]

Lipovsky et al.	The ability of broadband visible light illumination to promote proliferation of MSCs was evaluated.	[132]

Giannelli et al.	The effects of LLLT on mouse MSCs proliferation were investigated underlying cellular and molecular mechanisms, focusing the attention on the effects of laser irradiation on Notch-1 signal activation and membrane ion channel modulation.	[133]

Choi et al.	Adipose-derived mesenchymal stem cells- (ASCs-) seeded acellular dermal matrix was used with LLLT to repair bone defect.	[134]

Alexandrov et al.	Terahertz (THz) laser irradiation of MSCs can cause specific catalytic changes in cellular function that are closely related to the gene expression and differentiation state.	[135–137]

Wu et al.	The change in mRNA expression in rat MSCs after LLLT and the associated molecular mechanisms were investigated.	[138]

Wu et al.	LLLT induced IGF1 expression to promote both the proliferation and osteogenic differentiation of MSCs, whereas it may induce BMP2 expression primarily to enhance osteogenic differentiation.	[139]

Wang et al.	MicroRNA-193 proproliferation effects for bone MSCs were revealed after LLLT through inhibitor of growth family, member 5.	[140]

Soleimani et al.	The influence of LLLT at different energy densities on MSCs differentiation into neuron and osteoblast was examined.	[141]

Saygun et al.	LLLT increased the proliferation of osteoblast cells and stimulated the release of bFGF, IGF-1, and IGFBP3 from these cells.	[142]
